# Effects of communicating uncertainty descriptions in hazard identification, risk characterization, and risk protection

**DOI:** 10.1371/journal.pone.0253762

**Published:** 2021-07-13

**Authors:** Peter Wiedemann, Franziska U. Boerner, Frederik Freudenstein

**Affiliations:** 1 Jülich Research Centre, Jülich, Germany; 2 Australian Centre for Electromagnetic Bioeffects Research, Illawarra Health and Medical Research Institute, University of Wollongong, Wollongong, NSW, Australia; 3 Centre for Population Health Research on Electromagnetic Energy, Monash University, Melbourne, VIC, Australia; 4 School of Psychology, Faculty of Arts, Social Sciences & Humanities, University of Wollongong, Wollongong, NSW, Australia; 5 Institute of Occupational Medicine, Charité Universitätsmedizin Berlin, Berlin, Germany; 6 Department of Risk Communication, German Federal Institute for Risk Assessment (BfR), Berlin, Germany; 7 Department of Epidemiology and Preventive Medicine, School of Public Health and Preventive Medicine, Faculty of Medicine, Nursing and Health Sciences, Monash University, Melbourne, VIC, Australia; Fukushima Medical University School of Medicine, JAPAN

## Abstract

Uncertainty is a crucial issue for any risk assessment. Consequently, it also poses crucial challenges for risk communications. Many guidebooks advise reporting uncertainties in risk assessments, expecting that the audience will appreciate this disclosure. However, the empirical evidence about the effects of uncertainty reporting is sparse and inconclusive. Therefore, based on examples of potential health risks of electromagnetic fields (EMF), three experiments were conducted analysing the effects of communicating uncertainties separately for hazard identification, risk characterisation and risk protection. The setups aimed to explore how reporting and how explaining of uncertainty affects dependent variables such as risk perception, perceived competence of the risk assessors, and trust in risk management. Each of the three experiments used a 2x2 design with a first factor presenting uncertainty descriptions (as used in public controversies on EMF related health effects) or describing a certainty conditions; and a second factor explaining the causes of uncertainties (by pointing at knowledge gaps) or not explaining them. The study results indicate that qualitative uncertainty descriptions regarding hazard identification reduce the confidence in the professional competencies of the assessors. In contrast, a quantitative uncertainty description in risk characterisation–regarding the magnitude of the risk–does not affect any of the dependent variables. Concerning risk protection, trust in exposure limit values is not affected by qualitative uncertainty information. However, the qualitative description of uncertainty regarding the adequacy of protection amplifies fears. Furthermore, explaining this uncertainty results in lower text understandability.

## Introduction

There is a growing interest of risk researchers in conceptualizing and dealing with uncertainty [1–6]. One important question is how to communicate the uncertainties of a risk assessment to decision-makers and the public. Crucial is whether and to what extent uncertainties should be communicated and how this might affect the perception of the communicated risks [7]. From a normative point of view, it seems clear that uncertainties should be reported [8]. In its handbook on risk characterization, the United States Environmental Protection Agency (US EPA) [9] underlines that transparency is a principal value. It follows that describing uncertainties is an essential criterion for good risk characterization. Other scientific communities support this view too. For instance, global climate change researchers stress the need to communicate existing uncertainty beyond the scientific community to politicians, stakeholders, and the general public [10] and have proposed some standards for uncertainty communication [5].

Usually, a broadly shared assumption is that communicating uncertainty has positive effects on gaining trust and improving public confidence as seen in the WHO Outbreak communication guidelines [11, p.2]. Many social scientists support this view [12–14] or see only minor adverse effects [5]. However, others believe that communicating uncertainties can raise distrust in science [15, 16]. It follows that the issue of whether and how to communicate uncertainties is still unsettled.

### Communicating uncertainties

In the following, we will focus on three less addressed issues of uncertainty communication: (1) Does it matter to which aspect of the risk assessment the uncertainty refers? (2) Is the performative goal of uncertainty communication essential, i.e., whether the communications aim to amplify or attenuate risk perception? (3) What are the effects of explaining uncertainty?

In controversies about health effects of chemicals, but also other agents like non-ionising radiation, one key issue is the instrumentalization of uncertainty. Does an agent cause a detrimental health effect or not? This question is sometimes heavily disputed, and uncertainty used to amplify or to attenuate concerns. Similarly, numerical risk estimates are used to raise or to downplay public anxieties by highlight uncertainties or focusing worst-case scenarios. The same applies for risk management measures, as the controversies about value limits and precautionary action indicate [17]. With respect to these three issues, we can generalise Paul Slovic´s remark [18] and say that hazards, risks and management measures are battlefields. Therefore, we are interested in how normative assumptions like transparency of risk communication—which includes reporting uncertainties—may affect attitudes and beliefs of the general public. These insights could provide a first step in developing better risk communication, especially how to respond to the persuasive use of uncertainties for manipulative reasons.

#### Focus of uncertainty descriptions in risk assessments

Regarding risks, three main reference types of uncertainty can be distinguished: First, uncertainty referring to hazard identification (Is there a hazard?), second, uncertainty in risk characterization (How high is the risk?), and third, uncertainty in risk management (Is the proposed risk protection sufficient?) (see NRC 1994 [19]). In the following, these three types of uncertainties will be described in more detail using the examples of electromagnetic field (EMF) risk assessments. In doing so, we focus on how these uncertainties are described.

#### Uncertainty description about hazard identification

The distinction between hazard and risk is fundamental for risk assessment. Hazard means an "Inherent property of an agent or situation having the potential to cause adverse effects when an organism, system or (sub)population is exposed to that agent" [20, p. 12], while risk refers to the "probability of an adverse effect in an organism, system, or (sub)population caused under specific circumstances by exposure to an agent" [20, p. 13].

In many cases, it is uncertain whether a substance is a hazard. Therefore, hazard related uncertainty has to be described. In principle, there are two options. The uncertainty can be quantified or characterised qualitatively, i.e., by verbal phrases such as “the substance is probably carcinogenic to humans” or by similar hedging phrases. In the following we focus only on such verbal descriptions of uncertainty regarding hazards which are, for instance, used by the International Agency for Research on Cancer (IARC) [21].

To give one example: Whether exposure to radio-frequency electromagnetic fields (RF EMF) from cell phones below the limit values can cause an adverse health effect, such as cancer cannot be proved beyond a reasonable doubt. Not surprisingly, the IARC classified RF EMF and ELF (extremely low frequency) EMF as possibly carcinogenic to humans because some data support a cause-effect relation. However, the IARC highlights also that bias, confounders, and chance cannot be excluded with sufficient confidence [21]. Uncertainty, as this example clearly shows, refers here to hazard identification.

To our knowledge research on uncertainty communication has only marginally focused on uncertain hazards. However, there are studies on the interpretation of verbal uncertainty descriptions, which offer some opportunities and insights for studying uncertainty description about hazard identification [see 1]. The subjective meaning of verbal expression using probability or frequency indicators such as “probable”, “frequent”, “possible” or “often” differ from person to person [22], Furthermore, the laypersons’ understanding of verbal uncertainty categories does not necessarily correspond with the experts’ intended statement. Fischer and Jungermann [23] demonstrated that laypersons’ numerical equivalents of verbal labels (rarely, occasionally, and frequently) differ from the numerical definition given by the German Health Agency for these labels.

#### Uncertainty description about the risk characterization

Uncertainty can refer to risk characterization, i.e., on assessing the magnitude of the risk. This means that the existence of a hazard is proven (hazard identification) but the magnitude of the associated risk cannot be unambiguously determined. This type of uncertainty is indicated by confidence intervals with lower and upper bounds of the risk estimates.

Johnson and Slovic [24] showed that presenting a range of risk estimates increased risk perception compared to point estimates, usually the mean value. Han and colleagues provided a similar finding [25]. In another study of perception and judgment of uncertainties in risk estimations, Johnson and Slovic [24] observed that the upper limit of confidence intervals was viewed as the most reliable estimate. A study by Viscusi [26] supported this finding. Lofstedt et al. [27] found mixed effects. Glenton et al. [28] note that laypersons have difficulty understanding confidence intervals, i.e., they largely ignore or misunderstand those intervals.

Johnson [29] showed that a plurality attributes uncertainty expressed this type of uncertainty is usually attributed to social factors (primarily the self-interest and perceived incompetence of experts). When experts present uncertainties regarding their risk estimates, respondents were more likely to provide negative explanations (for instance, insufficient knowledge, intentional deceit).

Dieckmann et al. [30] explored the effects of uncertainty disclosure in forecasts on decision-makers and found somewhat positive effects. Decision-makers rated intelligence forecasts with ranges as higher in usefulness compared with forecasts based on point estimates if the probability of the forecasted event was high.

In a recent study by Howe et al. [31] on the perception of the effects of climate change on sea-level rise, the authors found that informing about the full range of possible effects (best and worst-case scenario) increased the acceptance of the "sea-level rise" message. However, when the addressees of the "full range" message were informed that the existing uncertainties could not be fully quantified, these positive effects disappeared.

It seems that there is no clear effect of quantitative uncertainty reporting by ranges. Maybe a look at heuristics in numerical cognition might help. First, we argue that in range perception a left number has an essential anchoring effect, similar the left digit-effect [32]. We assume that our propensity to read from left to right leads to putting more weight on the left number of two numbers that represent a range. A second option for explaining a different evaluation of range estimate versus a point estimate refers to the anchoring effect that describes the impact of initial number on the perception of subsequently given numbers [33]. Therefore, we postulate that adding a lower estimate of leukaemia cases to a given higher number of cases will lower risk perception.

#### Uncertainty about the adequacy of risk protection

Finally, uncertainties may relate to risk management. They pertain to the question of whether the risk management measures (e.g., the established limit values) are sufficient or not to prevent harm. For instance, one could argue that the most vulnerable people, such as children, pregnant women, or people suffering from chronic diseases, need extra protection that is not provided by the established value limits.

The effect of informing about the uncertainty concerning the adequacy of risk management measures—is to our knowledge—a little researched topic. Most research on exposure limits or standards is exploring why people trust or distrust in these protective measures. Only a few studies addressed the uncertainty of health protection by exposure limits. Wiedemann and Schütz [17] analysed the impact of uncertainty disclosure regarding risk protection messages by either adding or not adding a short paragraph about scientific uncertainty regarding the safety of the existing exposure limits. They found that expressing uncertainty about the sufficiency of risk protection does not affect risk perception. In a replication study, Wiedemann et al. [34] discovered no effects on risk perception and trust in public protection again.

### The performative goal of uncertainty communication

Uncertainty information can be framed in different ways and thereby used for different purposes. It can be used for raising fears and concerns or for reducing them. In other words, its performative force can have quite opposite goals.

Regarding hazard identification, uncertainty can be used either for questioning the existence of a hazard or for raising concerns that a hazard might exist. Concerning risk characterization, one can utilize uncertainty for suggesting that the risk might be low ("there is no conclusive evidence that a substantial risk exists"). Uncertainties can also be used to promote the view that the risk could be rather high ("even higher risks cannot be excluded".) Furthermore, regarding risk management, uncertainty can be applied for demonstrating that risk protection is insufficient. However, uncertainty could also be instrumentalized for claiming that risk protection is warranted by arguing that the risks underlying the protection measures are uncertain.

### Explaining uncertainty

Psychological research offers a broad range of insights into the types, formats, and functions of explanations in humans’ everyday thinking [35]. One insight is that explanations provide an interpretative context that shapes the understanding of an issue or happening. Therefore, it can be assumed that the explanation of uncertainty may affect how uncertainty is perceived and appraised. For example, the study participants of Morss et al. [36] preferred weather forecasts where uncertainty was explained over forecasts that indicated uncertainty without explanations.

However, the effects of explanation could depend on how uncertainties are explained. Smithson’s [37, 38] studies revealed that uncertainty is more tolerated if it is the product of inaccuracy than if it is caused by conflicting scientific views.

Regarding risk assessments, there are two options for the explanation of uncertainties. First, the explanation of uncertainty can refer to a lack of knowledge (epistemic uncertainty). Second, uncertainty can be declared as inherent, i.e., caused by random variation or stochastic behaviour of the risk-generating system [39]. This difference in explaining uncertainty matters because epistemic uncertainty could be used for blaming the risk assessor as incompetent. However, the risk assessor cannot be blamed for stochastic uncertainty [40].

However, it is not known how numeracy [41], health literacy [42], and risk literacy [43] impact the understanding of uncertainty explanations. These limited insights make it challenging to hypothesize how explaining uncertainty might affect risk perception, perceived competence of the assessors, and trust in risk management. Therefore, we refrain from formulating a hypothesis and instead favor an exploratory approach to explaining uncertainty.

### Research questions and hypotheses

Based on the above-discussed literature, we derived the following hypotheses:

It matters whether uncertainty is related to hazard identification, risk characterization, or risk management.

Whether information about uncertainties in hazard identification amplifies risk perception, depends on the performative force of uncertainty. If the baseline information (no uncertainties revealed) notes that a hazard exists, the disclosure of uncertainty in hazard identification that raises doubt that a hazard exists might reduce fears and risk perceptions.

If uncertainties refer to the risk magnitude expressed by a range of disease cases (lower limit versus upper limit of estimated childhood leukaemia cases) compared to a point estimate that indicates only the upper limit of the estimated leukaemia cases then the uncertainty information should elicit lower fears and risk perceptions. This is, because people who receive the range information got an additional anchor that will lower their perceptions.

If uncertainties refer to the adequacy of risk protection by risk management, i.e., if it is reported that the protection might not be established, fears and risk should increase compared to information about risk protection without uncertainties.

## Method

We conducted three experiments, each addressing a particular aspect of uncertainty reporting:

- Uncertainty description regarding hazard identification (R1)
- Uncertainty description about regarding risk characterization (R2)
- Uncertainty description regarding the adequacy of risk protection by risk management (R3)

The three experiments followed the same 2x2 factorial between-subjects design. The first factor U refers to the information about uncertainty (information about uncertainty versus no information about uncertainty). The second factor E addresses the explanation of uncertainty (presence of explanation versus no explanation of the uncertainty).

Factor U and Factor E are operationalized through different text modules. (see S1–S3 Textmodules). Table 1 sums up the main characteristics of the experimental variation.

**Table 1:** Main features of the experimental design.

	Used example	Certainty condition (basic information module)	Uncertainty condition	Format of uncertain-ty description	Explanation of uncertainty
R1	RF EMF exposure from cell phones	Hazard exists: "„radiation for cell phones is able to cause headaches “	Raising doubt about the existence of a hazard, i.e. hazard is uncertain (radiation from cell phones is likely to cause)	Verbal description	Reference to the knowledge gaps in hazard identification (epistemic uncertainty)
R2	ELF EMF exposure from high voltage power lines	The upper bound of the estimate of attributable leukaemia cases is given (2400 cases world-wide)	Informing about the uncertainty of the risk magnitude by giving the range of the attributable leukaemia cases (100–2400 cases worldwide)	Numerical range (lower and upper limit)	Reference to knowledge gaps regarding the number of children exposed to ELF EMF level > 0, 3 microtesla (epistemic uncertainty)
R3	RF EMF exposure from cell phones	Health protection is provided against proven risks	Raising doubt about the sufficiency of health protection (it cannot be conclusively determined, whether the limit values provide sufficient protection against possible but not scientifically proven long-term damages)	Verbal description	Reference to knowledge gaps of the existing value limits for RF EMF exposure (epistemic uncertainty)

Table 1 shows the different main characteristics of the experimental variations for R1 (hazard identification), R2 (risk characterization) and R3 (risk management).

In R1 (hazard identification), In R1 uncertainty description informs that the effect of RF EMF exposure on wellbeing is not proven, but likely. The baseline information used for estimating the impact of the uncertainty information on the dependent variables assumes that such a detrimental effect exists.

In R2 (risk characterization), uncertainty description informs about the range of potential childhood leukaemia cases due to EMF exposure from high voltage power lines. The baseline information used for estimating the impact of the uncertainty information on the dependent variables refers to a point estimate indicating only the upper bound of the range estimate.

In R3 (risk management), In R3 uncertainty description informs that health protection cannot be conclusively guaranteed. The baseline information used for estimating the impact of the uncertainty information on the dependent variables assumes that protection is established.

All the text modules used in the experiments are based on examples of real communications used by public health authorities and federal radiation protection agencies in Germany, Switzerland or the U.S. However, the original texts were simplified to match the expertise level of the participants (see S1 Table for a small selection of examples from public health authorities and agencies).

The dependent variables are measured on five different 7-point-Likert scales (see S1 and S2 Appendices), focusing on: (1) How understandable is the text? (2) Is the risk information clear and unambiguous? (3) Does the text raise doubts about the professional qualifications of the assessors? (4) What do you think is the magnitude of the risk described in the text? (5) Do you think the text raises fears? In the experiment R3, we employed an additional Likert scale measuring trust in risk protection (Do you trust that the exposure limit values protect health?).

At the time of the research data collected, social sciences research had not to be approved by an ethics committee at the Jülich research center. As the research in question was an experimental social science setup there participants were only asked to read different informational texts with no potential physical or psychological harmful information, no ethical approval was necessary at the time.

## Sample

For the experiments, we approached students from social science and humanities of the Technical University Dresden, the University of Bonn, and the RWTH Aachen, in Germany. Table 2 shows the further characteristics of this sample. The assignment of the study subjects to the treatment levels of the three experiments was randomized to reduce the impact of possible confounders. Randomization was conducted by a random number generator, which assigned the subjects to the four experimental treatment levels. Participants were asked to take part in the research after attending a lecture session. They were asked for their consent verbally and could leave anytime. Subjects had a debriefing after the experiment. The experiments were conducted in a face-to-face situation. In lieu of a participant information sheet a description of the study and information about what participation in the study involved was provided. The students had to read the short text and fill out the 5-item-questionnaire, taking about 5 minutes. Participants were free to refrain from participation and free to withdraw from the study at any time. Consent was obtained tacitly (via participation and completion of the survey) as indication of participants’ willingness to participate in the research. Participants received a debriefing after the experiment about the RF EMF case.

**Table 2:** Sample characteristics of the studies of hazard identification (R1), risk characterization (R2), risk protection by risk management (R3).

	Mean Age (range)	Gender (female)	Owns a mobile phone
R1 (N = 109)	22.3 (18–32)	65%	99%
R2 (N = 113)	22.1 (19–37)	61%	97%
R3 (N = 122)	23.1 (18–35)	59%	99%

## Statistics

For each of the three experiments R1, R2, and R3 we computed a 2x2 MANOVA with bootstrapping to examine the effects of the two factors "uncertainty" (U) and "explanation" (E) on the five dependent variables. The statistical analysis was conducted with SPSS 27. Group differences with p < 0.05 were marked as statistically significant. We used the Bonferroni- Holmes procedure for the adjustment of multiple testing [44]. In cases where the MANOVA requirements were not met, we applied the Welch test. The original dataset is provided as S1 Dataset.

## Results

### Effects of uncertainty description about information regarding hazard identification (R1)

Experiment R1 referred to the potential risks of reduced well-being caused by EMF exposure of cell phones. Uncertainty description informed that the effect of RF EMF exposure on wellbeing is not proven, but likely. The baseline information against which the uncertainty description was compared informed that adverse effects have been proven.

Table 3 informs about the effects of the experimental variation on the five dependent variables by providing means and the related confidence intervals.

**Table 3:** Effects of uncertainty information regarding hazard identification (R1).

Dependent variable	U0E0	U0E1	U1E0	U1E1
text understandability	M = 6,66	M = 6,51	M = 6,34	M = 6,18
	SD = 0.73	SD = 0.64	SD = 1.13	SD = 1.04
clarity of risk information	M = 5,85	M = 5,92	M = 4,65	M = 4,66
	SD = 1.68	SD = 1.36	SD = 1.94	SD = 1.80
doubts in the professional competencies of the responsible experts	M = 4,37	M = 3,55	M = 4,84	M = 5,03
	SD = 1.57	SD = 1.83	SD = 1.40	SD = 1.50
risk perception	M = 3,55	M = 4,14	M = 3,38	M = 3,77
	SD = 1.60	SD = 1.56	SD = 1.55	SD = 1.22
fear arousal	M = 2,81	M = 3,63	M = 3,27	M = 2,67
	SD = 1.81	SD = 1.57	SD = 2.03	SD = 1.11

Means (M) of the dependent variables, measured on 7-points Likert scales (the higher the scores, the higher the value on the variables) and standard deviations (SD) across the four treatment levels in experiment R1(hazard identification).

U0 = no uncertainty, U1 = uncertainty revealed, E0 = no explanation, E1 = explanation given.

As Table 3 reveals that the understandability of the text modules is judged as high. The same applies for the clarity of the given information. However, here lower scores for the uncertainty conditions (U1E0, U1E1) are evident. Doubts regarding the technical competence of the experts who conducted hazard identification are in a medium range. Interestingly, risk perception is not particularly elevated, and the fear level is rather low.

**Fig 1:**
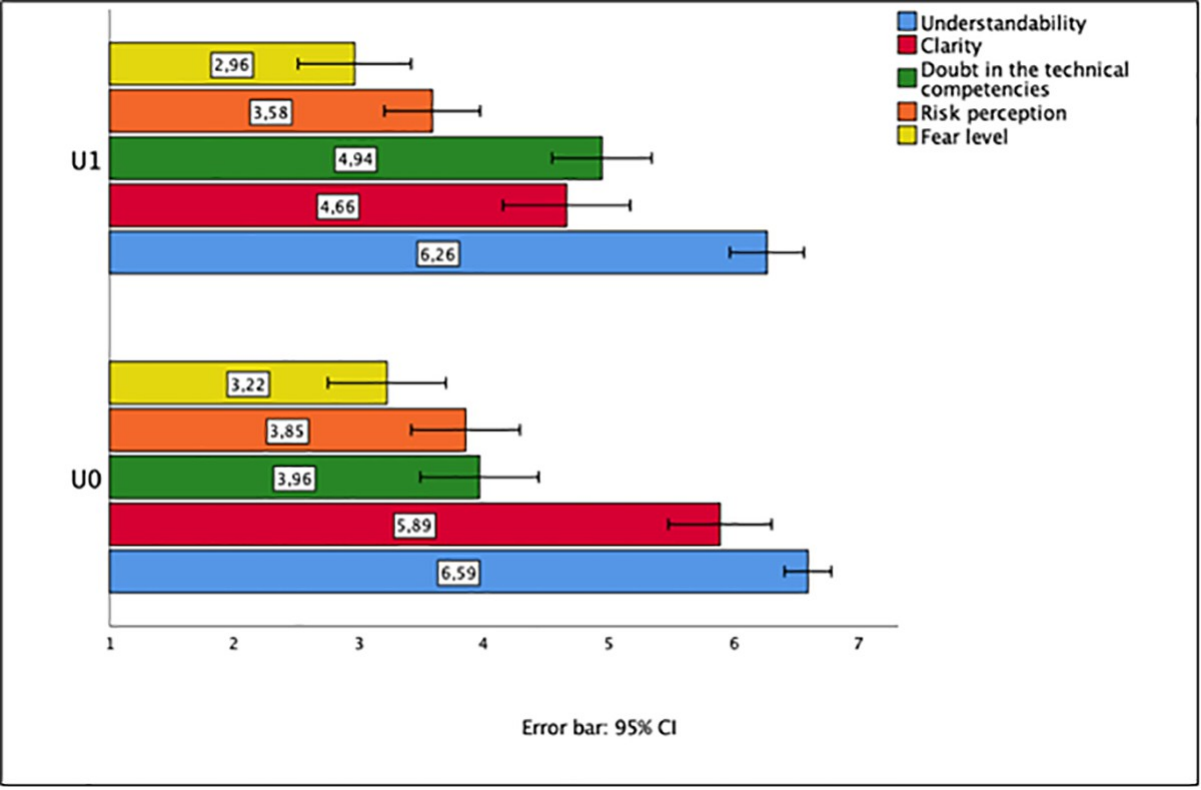
Effects of uncertainty description about regarding hazard identification (factor uncertainty) (R1). Means (with 95% confidence intervals) of the dependent variables (measured on 7-points-Likert scales) for the two levels of factor “uncertainty—U0: no information about uncertainty, U1: information about uncertainty). N.B.: The higher the score on the Likert scale, the higher is the value on the dependent variable.

A two-factor MANOVA (factors „uncertainty" and „explanation") on the five dependent variables revealed that the error variances are not homogeneous, as assessed by Levene’s test (p < 0.05). Therefore, we computed for each factor the Welch test on the five independent variables. For the factor „uncertainty" the test provided the following results: There are statistically significant effects on the variables "clarity of information" and "doubts about the professional qualification of the assessors" (F(1,101) = 16.04, p< 0.0001, η^2^ = 0.131, and F(1,105) = 10.07, p< 0.002, η^2^ = 0.087). When uncertainty refers to hazard identification ("RF EMF exposure is likely to cause detrimental health effects"), information is judged to be less clear compared to an information indicating that RF EMF causes detrimental health effects. In addition, uncertainty information raises more doubt about the professional competencies of the risk assessors compared to information about hazard identification without uncertainty disclosure (info: “RF EMF exposure can cause detrimental health effects”). All other variables show no statistically significant effects. Fig 1 depicts the effects of the given qualitative "uncertainty" information on the dependent variables.

The Welch test on the second experimental factor "explanation" does not indicate any significant effect on the dependent variables. However, concerning the factor "explanation" we are primarily interested whether the explanation of uncertainty in hazard identification affects the five dependent variables. Therefore, we restricted our analysis on the relevant data subset, i.e., on the two experimental groups U1E0 (no explanation of uncertainty) and U1E1 (explanation of uncertainty). Because the Levene test indicated that the variances are not homogeneous we applied the Welch test, which provided no statistically significant effects (for all dependent variables applies: F(1,54) = 2.144, p > 0.151). In other words, explaining the uncertainty of hazard identification (explaining why the hedging phrase “likely” is used) by informing about knowledge gaps did not statistically significant affect the five dependent variables.

Our hypotheses that describing uncertainty about EMF hazard identification reduces risk perception and fear is not supported by our findings. However, we were able to show that uncertainty information raised doubt about the professional competencies of the experts who conducted the assessment, and it decreased the understandability of the given information. Furthermore, an explanation of uncertainty by referring to knowledge gaps had no statistically significant effects on the dependent variables.

### Effects of uncertainty description about regarding risk characterization (R2)

Experiment R2 referred to the potential risks of reduced well-being caused by EMF exposure of power lines. Uncertainty description informed about the range (lower and upper estimate) of potential childhood leukaemia cases due to EMF exposure. The baseline information against which the uncertainty description was compared referred to a point estimate indicating only the upper bound of the range estimate.

Table 4 indicates that mean scores for text understandability and clarity of risk information is evaluated as rather medium. The same applies to doubts in the professional competencies of the risk assessors. Furthermore, the mean scores for doubts in the professional competencies of the assessors, risk perception and fear arousal are almost all below the scale mid points indicating low concerns.

**Table 4:** Effects of uncertainty description about risk characterization (R2).

Dependent variable	U0E0	U0E1	U1E0	U1E1
text understandability	M = 5.04	M = 4,75	M = 4.85	M = 4.72
	SD = 1.62	SD = 1.60	SD = 1.48	SD = 1.39
clarity of risk information	M = 4.44	M = 4.46	M = 4.81	M = 4.28
	SD = 1.55	SD = 1.6	SD = 1.55	SD = 1.78
doubts in the professional competencies of the responsible experts	M = 3.89	M = 3.54	M = 3.73	M = 3.88
	SD = 1.45	SD = 1.34	SD = 1.69	SD = 1.66
risk perception	M = 3.56	M = 4.25	M = 3.69	M = 3.44
	SD = 1.48	SD = 1.32	SD = 1.23	SD = 1.44
fear arousal	M = 2.96	M = 3.46	M = 3.19	M = 2.75
	SD = 1.99	SD = 1.45	SD = 1.65	SD = 1.44

Means (M) of the dependent variables, measured on 7-points Likert scales (the higher the scores, the higher the value on the variables), and standard deviations (SD) across the four treatment levels in experiment R2 (risk characterization).

U0 = no uncertainty, U1 = uncertainty revealed, E0 = no explanation, E1 = explanation given.

For testing our hypotheses on the effects of informing about uncertainty and explaining uncertainty in risk characterization, we computed a 2-factor MANOVA with the five dependent variables. The Levene test indicated the homogeneity of the error variances (p > 0.05). There is also a homogeneity of covariances, as assessed by Box’s test (p = 0.168).

The MANOVA revealed no statistically significant effects of both factors uncertainty and explanation (for all dependent variables applies: F(1,109) = 1.701, p > 0.5). There are also no statically significant interaction effects (for all dependent variables applies: F(1,109) = 3.36, p > 0.7). The results show, that there are no differences, no matter whether information about uncertainty is provided (i.e, the full range of estimates) or not (i.e, only the upper limit of the estimated number of childhood leukaemia cases) and whether explanations for the uncertainty are given or not. This does not have any effect on the subjective evaluation of the understandability and the unambiguousness of the information. Likewise, it does not influence the subjective assessment of the magnitude of the risk or on the feelings of fear, nor does it cast any doubts on the professional competence of the risk assessors.

It remains to be noted that our findings don’t support our hypothesis about disclosing uncertainty about risk characterization. Giving the lower and the upper estimated number of leukaemia cases—compared to informing only about the upper estimate—reduced either risk perception nor fear-arousal. In addition, explaining uncertainties by referring to knowledge gaps in risk characterization had no statistically significant effects on the dependent variables.

### Effects of uncertainty description about protection by risk management (R3)

The experiment R3 referred to the potential risks of childhood leukaemia by EMF exposure of cell phones. Uncertainty description informed that health protection cannot be conclusively guaranteed. The baseline information against which the uncertainty description was compared highlighted that protection has been established.

Table 5 provides information about the dependent variables’ means for the four treatment levels of experiment R3. Note that for this setup an additional variable, labelled "trust in risk protection" was added. The means of text understandability are above the scale midpoint, but the clarity of information is somewhat lower and centres about the scale midpoint. The same is true for the scores on the scales referring to "doubt in the professional competence", "risk perception", and "trust on risk protection". Only the means for fear arousal are clearly below the midpoint of the scale.

**Table 5:** Effects of uncertainty description about protection by risk management (R3).

Dependent variable	U0E0	U0E1	U1E0	U1E1
text understandability	M = 6.26	M = 5.52	M = 6.00	M = 4.83
	SD = 1.11	SD = 1.55	SD = 1.10	SD = 1.61
clarity of risk information	M = 3.9	M = 3.55	M = 4.17	M = 4.26
	SD = 1.96	SD = 1.90	SD = 1.63	SD = 1.74
doubts in the professional competencies of the responsible experts	M = 3.93	M = 4.00	M = 3.71	M = 4.52
	SD = 1.79	SD = 1.84	SD = 1.49	SD = 1.44
risk perception	M = 3.93	M = 3.76	M = 3.87	M = 3.74
	SD = 1.70	SD = 1.41	SD = 1.51	SD = 1.36
trust in risk protection	M = 3.86	M = 3.76	M = 3.92	M = 4.22
	SD = 1.80	SD = 1.83	SD = 1.50	SD = 1.35
fear arousal	M = 1.81	M = 2.45	M = 2.83	M = 2.52
	SD = 1.11	SD = 1.50	SD = 1.44	SD = 1.47

Means (M) of the dependent variables, measured on 7-points Likert scales (the higher the scores, the higher the value on the variables), and standard deviations (SD) across the four treatment levels in experiment R3 (risk management).

U0 = no uncertainty, U1 = uncertainty revealed, E0 = no explanation, E1 = explanation given.

For testing our hypotheses on the effects of informing and explaining uncertainty regarding risk protection, we computed a 2-factorial MANOVA with the six dependent variables. The Levene test indicated the homogeneity of the error variances (p > 0.05). There was also a homogeneity of covariances, as assessed by Box’s test (p = 0.103).

**Fig 2:**
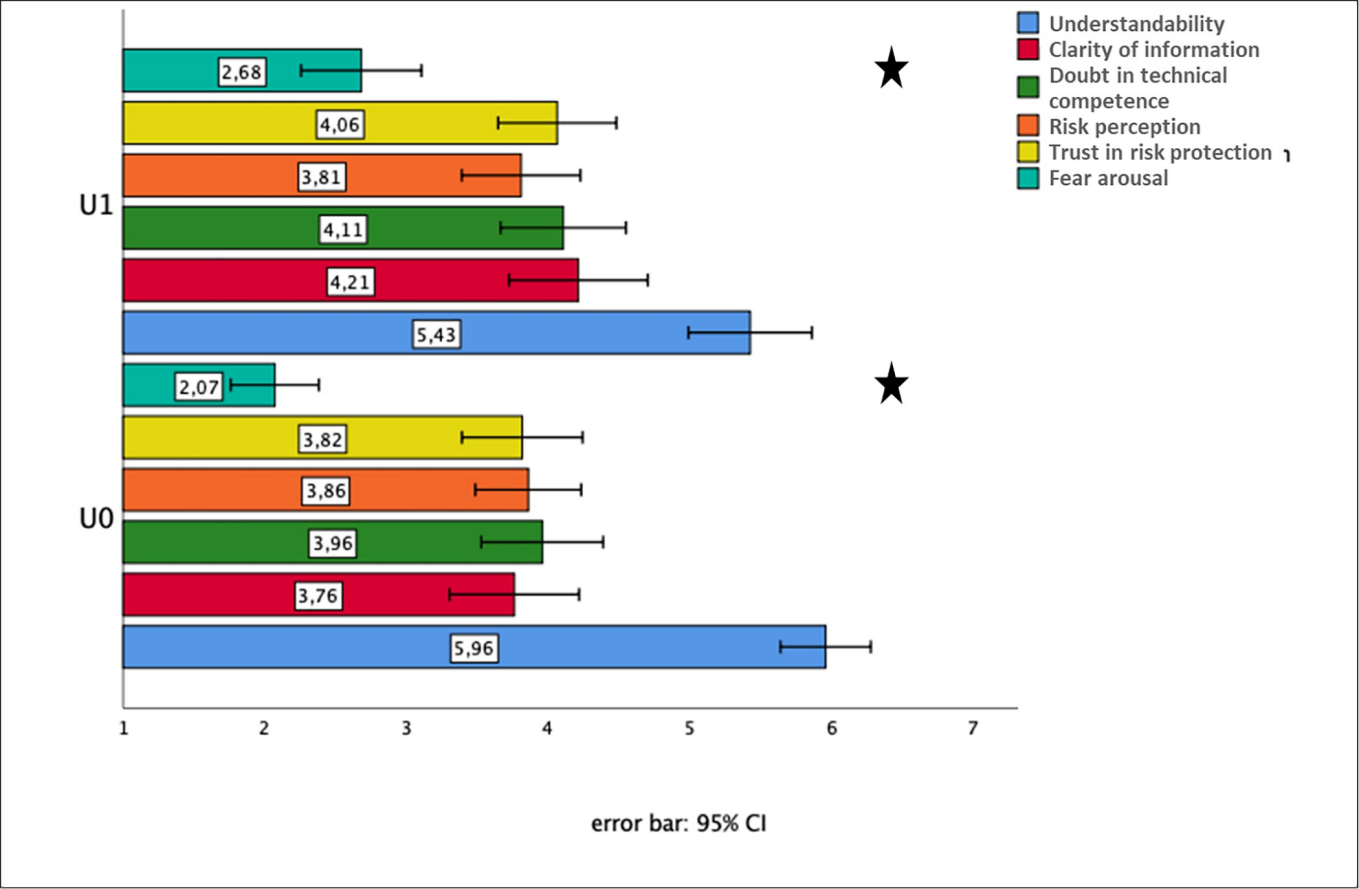
Effects of uncertainty description about protection by risk management (factor uncertainty) (R3). Means (with 95% confidence intervals) of the dependent variables (measured on 7-point-Likert scales) for the two levels of factor “uncertainty”—U0: no information about uncertainty, U1: information about uncertainty. N.B.: The higher the score on the Likert scale the higher is the value on the dependent variable.

For the factor uncertainty, our data indicate a statistically significant effect for fear arousal (see Fig 2). If uncertainty about protection by risk management is presented in a qualitative format, then the text aroused more fear (F(1,114) = 4.6, p < 0.05, partial η^2^ = 0.039). With respect to all other dependent variables, we did not observe any statistically significant effects (all p> 0.05). Especially, presenting uncertainty about protection by risk management does not influence risk perception and trust in risk protection regarding the established exposure value limits, nor does it increase doubts on the professional qualification of the risk assessors.

**Fig 3:**
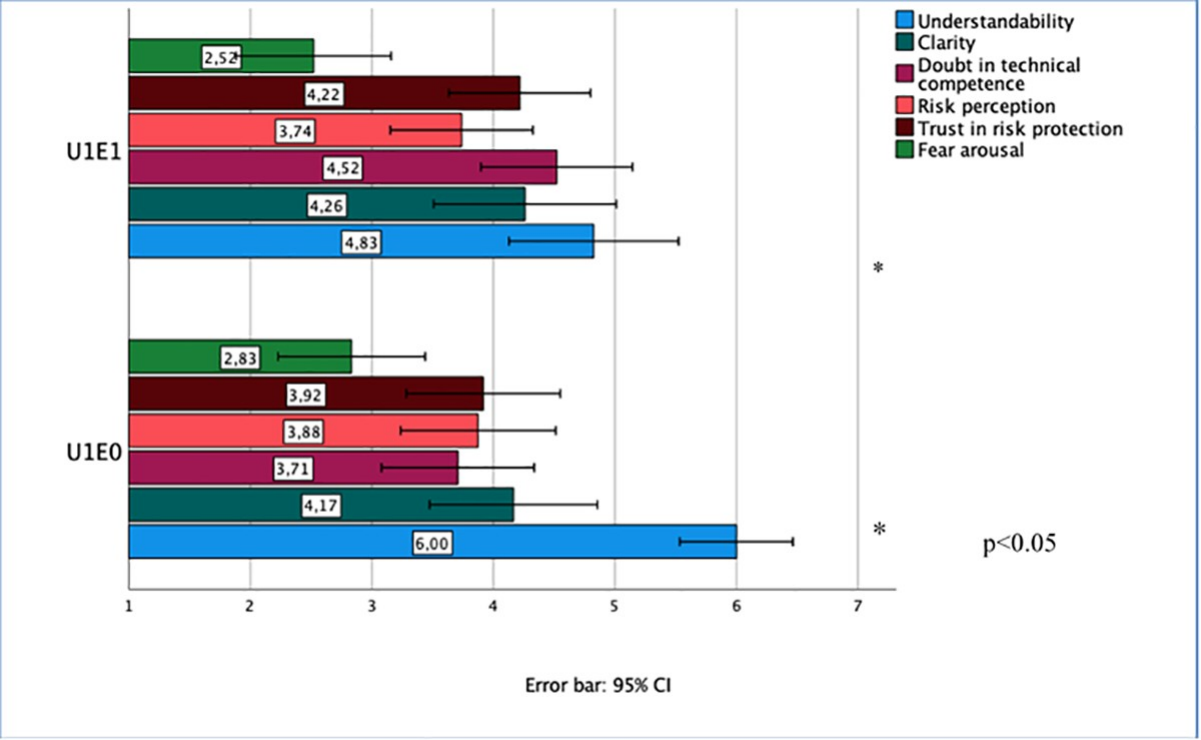
Effects of uncertainty explanation about protection by risk management (factor explanation) (R3). Means (with 95% confidence intervals) of the dependent variables (measured on 7-point Likert scales) for the two levels of factor “explanation”—U1E0: uncertainty, no explanation, U1E1: uncertainty, explanation, N.B.: The higher the score on the Likert scale the higher is the value on the dependent variable.

The factor explanation provides only one statistically significant result that refers to text understandability (see Fig 3). If risk management measures are explained, whether or not information about uncertainty is provided, the understandability of the text is reduced (F(1,114) = 14.435, p < 0.001, partial η^2^ = 0.034).

Because we are above all interested in the effects of explaining uncertainty, we compared solely the data set U1E0 and U1E1 as a subset. An ANOVA delivered one statistically significant result. If the uncertainty about risk protection by risk management is explained by indicating knowledge gaps, the text understandability is reduced (F(1,47) = 5,005, p < 0.05, η^2^ = 0.097). All other dependent variables remained unaffected by the given explanation.

To summarize, the findings of experiment R3 indicate that the qualitative characterization of uncertainty about protection by risk management using EMF exposure limits leads to statistically significant increased fear arousal. However, there were no amplified doubts about the health protection by the established EMF exposure limits. Therefore, the findings of experiment R3 do not support our hypothesis, which expected an increase in risk perception and fear arousal. Furthermore, explaining uncertainty by referring to knowledge gaps affected statistically significantly only the text understandability, which decreased in size.

## Discussion

For the first time, this study separately analysed the effects of uncertainty descriptions about hazard identification, risk characterization, and risk management using examples from the EMF debate. Regarding hazard identification we focused on EMF exposure from power lines and used qualitative terms for describing uncertainty. The uncertainty information highlighted that detrimental effects of EMF exposure on well-being are not established, but likely. In the ‚certainty’ condition experimental subjects were informed that these detrimental effects are established.

The findings of the experiment R1 did not support our hypothesis that uncertainty description which puts the existence of a hazard into question would reduce risk perception and fear arousal. A possible explanation would be that non-experts like our study participants do not differentiate between effects that are likely and those which are confirmed. Furthermore, there could be an impact of prior beliefs on risk and fear arousal that masks the expected effects [45].

Special interest deserves the impact on perceived competence of the assessors. Information about uncertainty regarding hazard identification can be misattributed as a sign of an incompetent assessment. This finding contradicts with other studies that found a positive effect of disclosing uncertainty [46]. However, our findings are in line with van der Bles et al. [47]. They found that verbal uncertainty reporting impaired people’s trust in the given numerical information compared with the control condition (no uncertainty information) and the reporting of numerical uncertainty.

Furthermore, in our it turned out that reporting uncertainty by verbal means makes information less clear. This is understandable, taken into account that uncertainty reporting raises complexity and most lay persons usually prefer simple, clear cut evaluations [see 48].

Qualitative uncertainty characterization, which is used by IARC [21], EFSA [49] and other agencies responsible in hazard identification, provides some challenges regarding perceived professional competencies. However, further research should clarify whether losing trust in these competencies is compensated by gaining social trust due to openness and transparency of risk communication.

In experiment R2, we referred to the childhood leukemia issue, which is most prominent in the debate on the detrimental effects of EMF exposure by power lines. Focusing on risk characterization, we used a quantitative description of uncertainty. In the uncertainty condition, study participants were informed that worldwide 100 to 2400 childhood leukemia cases per year could be attributed to EMF emissions from power lines. In the certainty condition, subjects were only informed about the upper bound of the range estimate, i.e., EMF exposure might cause worldwide 2400 cases of childhood leukemia per year. Most importantly, our findings indicate that reporting uncertainty by ranges has no statistically significant effect on any of the dependent variables compared with a point estimate that presents the upper bound of the estimates. It might be possible that the absence of an impact on risk perception and fear is caused by the negativity bias [50], i.e., by the tendency to pay more attention to worst-case information. In other words, our results could be explained by an upwards bias. However, it is also possible that a confirmation bias affected our findings. Respondents who already believe that EMF causes harm tend to prefer information that fits their beliefs (see Kunda [51], for a general discussion), in our case, the worst-case estimate.

Experiment R3 referred to health protection relating to the potential risks of EMF exposure from cell phones. Here, we described uncertainty by qualitative terms. In the uncertainty condition, subjects were informed that the limit values would protect humans against the scientifically proven risks caused by electromagnetic fields. However, they were also informed that it could not be conclusively decided whether the limit values also provide sufficient protection against possible, but not scientifically proven, long-term health effects. In the certainty condition, this additional information was not provided,

Our hypothesis that uncertainty about the adequacy of risk management amplifies risk perception and fear found only partially support. In the uncertainty condition, only fear increased statistically significant. Contrary to our expectation, uncertainty did not affect trust in the adequacy of risk management measures.

Furthermore, explaining uncertainty had virtually no effect, with only one exception. Explaining uncertainty of health protection reduced the text’s understandability because it adds more complexity to the text. Also, our findings do not support the assumption that explaining uncertainty in hazard identification by indicating knowledge gaps is judged negatively by laypersons.

However, we recommend our readers to consider our findings of explaining uncertainty in risk assessments with some reservations. First, the topic ‚explanation`is a broad field with contributions from various research communities, e.g., linguistics, cognitive psychology, and education sciences. We are dealing in our experiment only with a tiny aspect, explaining uncertainties in risk hazard identification, risk characterization, and risk management. Second, even in this narrow field, our explanatory text modules, although based on explanations given by organizations responsible for radiation protection in the German-speaking countries, could have been structured differently, in wording, length and complexity. Third, our wording nudged the study participants to attribute the uncertainty to the risk assessors’ lack of expertise. It may well be that triggering the attribution of uncertainty to complexity could have led to a different result.

Therefore, our results are bounded to the chosen modules. Because of this, the absent impact of explaining uncertainties should be by no means taken for granted. Other text modules could have had different results. In our opinion, it requires more explorative research before the effects of explaining uncertainty can be a topic of hypothesis-driven experimental research.

### Limitations

Our findings have several limitations. First of all, the bias of hasty generalisation should be avoided. Our results are bounded on a specific issue—potential EMF related health risks, particular operationalisation and an ad hoc sample of rather well-educated subjects.

Furthermore, the choice of health endpoints could matter. For instance, the endpoint that was used in experiment R1 (hazard identification) referred to impairments of well-being, i.e., headaches. With another endpoint, e.g., brain cancer, the experiment could have provided other findings.

In addition, we assume that research on uncertainty in risk assessment requires to focus on inter-individual differences. It seems that the specific characteristics of target groups needs more attention, especially risk literacy and numeracy. To give one example: The communication of ranges could lead to quite different perceptions. Depending on a-priori beliefs and attitudes, people might prefer either the lower or the upper bound of the range as best risk estimate. So far, there are no evidence-based guidelines on how to deal with this issue.

Furthermore, our findings do not rule out that informing about uncertainty in risk management, could reduce trust in heath protection. A more affective description of the health consequences of lack of protection, e.g., possible cancerous diseases, could have yielded amplified risk perceptions.

The results to the second factor explanations are at best preliminary, as they are bound to the chosen operationalization of our text modules. Furthermore, one may critique the use of unvalidated risk perception scales. This issue has been discussed in Wilson et al. [52] and is a widely known phenomena in the risk perception research field. However, up until know, there is no standardized scale to measure intuitive risk perception available.

## Conclusions

Our experiments provide several implications for reporting uncertainties. They show that informing about uncertainties is not always a means for improving trust and credibility. Science skeptics and activists with a particular political agenda might instrumentalize uncertainty for spreading distrust in science, primarily when uncertainty refers to hazard identification. Risk communicators should be aware that admitting uncertainty is a double-edged sword. Laypersons might attribute it to the lack of professional expertise.

Further research should focus on the issues of cognitive resources and prior beliefs. It should focus on how the recipients interpret the specific social context in which the information is given and how the recipients’ cognitive resources, motivations to process information, and their prior beliefs influence uncertainty information interpretation. These issues could be tackled by dual-process theories developed in judgment and decision-making [53]. Recipients might understand the same information differently depending on whether the information is processed by elaborative or heuristic cognition. Furthermore, prior beliefs shape the interpretation of uncertainties in risk assessment. This issue can be tackled by theories that explain how motivated reasoning influences judgments and opinions about uncertainty information [54].

Finally, we would like to stress the importance of the performative goal of informing about uncertainty. It makes a massive difference whether uncertainty is used to raise fears and distrust in science or honestly report the limits of evidence-based knowledge. Further research should pay more attention to this subject. New studies could learn from the analysis of the politicization of science [16] that has focused on how uncertainty is exploited for undermining trust in science.

## Supporting information

S1 Appendix Questions and response scales. (PDF)

S2 Appendix Questionnaire with text module. (PDF)

S1 Textmodule Text vignettes for experiment R1. (PDF)

S2 Textmodule Text vignettes for experiment R2. (PDF)

S3 Textmodule Text vignettes for experiment R3. (PDF)

S1 Table Examples for communicating uncertainty in the EMF area. (PDF)

S1 Dataset (SAV)
