# Supplementary material for: Effects of communicating uncertainty descriptions in hazard identification, risk characterization, and risk protection
Source: PLoS One. 2021 Jul 13;16(7):e0253762. doi: 10.1371/journal.pone.0253762 (PMC8277037; doi:10.1371/journal.pone.0253762)
Supplement: S1 Appendix — (PDF) [file pone.0253762.s001.pdf]

S1 Appendix: Questions and response scales (translated from German).

**How understandable is the text?**		
Not at all understandable	□ □ □ □ □ □ □	Very understandable
**Is the risk information clear and unambiguous?**		
Not at all clear & unambiguous	□ □ □ □ □ □ □	Very clear & unambiguous
**Does the text raise doubts for the technical qualifications of the risk assessors?**		
Absolutely no doubt	□ □ □ □ □ □ □	A lot of doubt
**What do you think is the magnitude of the risk described in the text?**		
No risk at all	□ □ □ □ □ □ □	Very large risk
**Do you think the text raises fears?**		
Not at all	□ □ □ □ □ □ □	Yes, very much so
