# Supplementary material for: Effects of communicating uncertainty descriptions in hazard identification, risk characterization, and risk protection
Source: PLoS One. 2021 Jul 13;16(7):e0253762. doi: 10.1371/journal.pone.0253762 (PMC8277037; doi:10.1371/journal.pone.0253762)
Supplement: S2 Appendix — (PDF) [file pone.0253762.s002.pdf]

S2 Appendix: Questionnaire with text module (translated from German).

Questionnaire for Study

Location: ________________________________

Date: [TT/MM/JJJJ]

Socio-demographic information about the participant

Gender (please check):

- ☐ female
- ☐ male

Age: _______ Years

University Subject: ____

The present study is part of a study that is being carried out on behalf of the Federal Office for Radiation Protection and which serves to improve the information offered by the Federal Office. Please read the following text module carefully and answer the related questions. Follow your subjective impression - there are no right or wrong answers. We are interested in your personal evaluation.

Textmodule to be assessed:

There is a connection between magnetic field radiation of the power supply over 0.3 microtesla and leukemia diseases (blood cancers) in children.<br><br>This means that up to 2400 childhood leukaemia diseases can be expected worldwide every year due to the magnetic field radiation of the power supply. That is about 5% of the 49,000 leukaemia cases occurring worldwide each year.<br><br>Several large risk studies that have summarized the available data on childhood leukemia come to this conclusion.

Please tick a box according to your assessment.

How understandable do you find the text?		
Not at all understandable	☐ ☐ ☐ ☐ ☐ ☐ ☐	Very understandable

Please tick a box according to your assessment.

Is the risk information clear and unambiguous?		
Not at all clear & unambiguous	☐ ☐ ☐ ☐ ☐ ☐ ☐	Very clear & unambiguous

Please tick a box according to your assessment.

Does the text raise doubts about a professionally qualified risk assessment?		
Absolutely no doubt	☐ ☐ ☐ ☐ ☐ ☐ ☐	A lot of doubts

Please tick a box according to your assessment.

How great do you think the risk described in the text is?		
No risk at all	☐ ☐ ☐ ☐ ☐ ☐ ☐	Very large risk

Please tick a box according to your assessment.

Do you find the text scary?		
Not at all	☐ ☐ ☐ ☐ ☐ ☐ ☐	Yes, very much so

Please tick a box according to your assessment.

How do you rate the text in terms of the following categories?		
interesting	☐ ☐ ☐ ☐ ☐ ☐ ☐	boring
too short	☐ ☐ ☐ ☐ ☐ ☐ ☐	too extensive
easy to understand	☐ ☐ ☐ ☐ ☐ ☐ ☐	difficult to understand
coherent	☐ ☐ ☐ ☐ ☐ ☐ ☐	incoherent
few unfamiliar words	☐ ☐ ☐ ☐ ☐ ☐ ☐	many unfamiliar words
good distinction between the essential and non-essential	☐ ☐ ☐ ☐ ☐ ☐ ☐	lacking distinction between the essential and the non-essential
simple sentences	☐ ☐ ☐ ☐ ☐ ☐ ☐	overloaded sentences
concrete	☐ ☐ ☐ ☐ ☐ ☐ ☐	abstract
clear	☐ ☐ ☐ ☐ ☐ ☐ ☐	confusing

fluent	☐ ☐ ☐ ☐ ☐ ☐ ☐	awkward
vivid	☐ ☐ ☐ ☐ ☐ ☐ ☐	indistinguishable
easy	☐ ☐ ☐ ☐ ☐ ☐ ☐	complicated
memorable	☐ ☐ ☐ ☐ ☐ ☐ ☐	hard to remember
limited to the essentials	☐ ☐ ☐ ☐ ☐ ☐ ☐	a lot of irrelevant things

If you have any further comments on the text module, please write them down here:
