# Supplementary material for: Effects of communicating uncertainty descriptions in hazard identification, risk characterization, and risk protection
Source: PLoS One. 2021 Jul 13;16(7):e0253762. doi: 10.1371/journal.pone.0253762 (PMC8277037; doi:10.1371/journal.pone.0253762)
Supplement: S1 Textmodule — (PDF) [file pone.0253762.s003.pdf]

S1 Textmodule: Text vignettes for experiment R1.

Version	Textmodule
No uncertainty<br>No explanation	*Radiation emitted from mobile phones is able to cause headaches, tiredness, difficulties in concentration, discomfort, and burning sensation on the skin.*
No uncertainty<br>Explanation	*Radiation emitted from mobile phones is able to cause headaches, tiredness, difficulties in concentration, discomfort, and burning sensation on the skin. Scientific studies prove this causal relationship. The negative health effect can be directly attributed to the radiation. They cannot be accounted for by other associated factors of mobile communication.*
Uncertainty<br>No explanation	*It is likely that radiation emitted from mobile phones is able to cause headaches, tiredness, difficulties in concentration, discomfort, and burning sensation on the skin.*
Uncertainty<br>Explanation	*It is likely that radiation emitted from mobile phones is able to cause headaches, tiredness, difficulties in concentration, discomfort, and burning sensation on the skin. Scientific studies, however, cannot prove this causal relationship. The negative health effects cannot be directly attributed to the radiation. They could also be accounted for by other associated factors of mobile communication.*
