# Supplementary material for: Effects of communicating uncertainty descriptions in hazard identification, risk characterization, and risk protection
Source: PLoS One. 2021 Jul 13;16(7):e0253762. doi: 10.1371/journal.pone.0253762 (PMC8277037; doi:10.1371/journal.pone.0253762)
Supplement: S2 Textmodule — (PDF) [file pone.0253762.s004.pdf]

S2 Textmodule. Text vignettes for experiment R2.

Version	Textmodule
No uncertainty, no explanation	There is causal relationship between EMF emissions from power lines above 0,3 microtesla and childhood leukaemia. Consequently, about 2400 worldwide cases of childhood leukaemia can be attributed to EMF emissions from power lines. This represents about 5% of the worldwide 49,000 annual leukaemia incidences.
No uncertainty, explanation	There is a causal relationship between EMF emissions from power lines above 0,3 microtesla and childhood leukaemia. Consequently, about 2400 worldwide cases of childhood leukaemia can be attributed to EMF emissions from power lines. This represents about 5% of the worldwide 49,000 annual leukaemia incidences. Several large-scale risk studies that have integratively evaluated the available data concerning childhood leukaemia have reached the same conclusion.
Uncertainty No explanation	There is a causal relationship between EMF emissions from power lines above 0,3 microtesla and childhood leukaemia. Consequently, between 100 and 2400 worldwide cases of childhood leukaemia can be attributed to EMF emissions from power lines. This represents between 0.2% - 5% of the worldwide 49,000 annual leukaemia incidences.

Uncertainty Explanation	There is a causal relationship between EMF emissions from power lines above 0,3 microtesla and childhood leukaemia. Consequently, between 100 and 2400 worldwide cases of childhood leukaemia can be attributed to EMF emissions from power lines. This represents between 0.2% - 5% of the worldwide 49,000 annual leukaemia incidences. Several large-scale risk studies that have integratively evaluated the available data concerning childhood leukaemia have reached the same conclusion. The exact number of possible leukaemia cases can not be determined because it is not known how many children are exposed to magnetic fields above 0,3 microtesla.
