# Supplementary material for: Effects of communicating uncertainty descriptions in hazard identification, risk characterization, and risk protection
Source: PLoS One. 2021 Jul 13;16(7):e0253762. doi: 10.1371/journal.pone.0253762 (PMC8277037; doi:10.1371/journal.pone.0253762)
Supplement: S3 Textmodule — (PDF) [file pone.0253762.s005.pdf]

S3 Textmodule: Text vignettes for experiment R3.

Version	Textmodule
No uncertainty<br>No explanation	For the protection of the public the “Ordinance on electromagnetic fields - 26. BImSchV” has been enacted on 16.12.1996 in Germany. This regulation sets limit values for health protection. Compliance with these limit values will protect humans against the scientifically proven risks that can be caused by electromagnetic fields.
No uncertainty<br>Explanation	For the protection of the public the “Ordinance on electromagnetic fields - 26. BImSchV” has been enacted on 16.12.1996 in Germany. This regulation prescribes limit values for health protection. Compliance with these limit values will protect humans against the scientifically proven risks that can be caused by electromagnetic fields.<br>This assessment is based on the careful evaluation of all available scientific studies that have looked for health risks from mobile communication.
Uncertainty<br>No explanation	For the protection of the public the “Ordinance on electromagnetic fields - 26. BImSchV” has been enacted on 16.12.1996 in Germany. This regulation prescribes limit values for health protection. Compliance with these limit values will protect humans against the scientifically proven risks that can be caused by electromagnetic fields. However, it cannot be conclusively determined, whether the limit values also provide sufficient protection against possible, but yet not scientifically proven long-term damages.

Uncertainty Explanation	For the protection of the public the “Regulation of electromagnetic fields - 26. BImSchV” has been enacted on 16.12.1996 in Germany. This regulation prescribes limit values for health protection. Compliance with these limit values will protect humans against the scientifically proven risks that can be caused by electromagnetic fields. However, it cannot be conclusively determined, whether the limit values also provide sufficient protection against possible, but yet not scientifically proven long-term damages. This assessment is based on the careful evaluation of all available scientific studies that have looked for health risks from mobile communication. They show that there are knowledge gaps regarding long-term effects for humans.
