# Supplementary material for: Effects of communicating uncertainty descriptions in hazard identification, risk characterization, and risk protection
Source: PLoS One. 2021 Jul 13;16(7):e0253762. doi: 10.1371/journal.pone.0253762 (PMC8277037; doi:10.1371/journal.pone.0253762)
Supplement: S1 Table — (PDF) [file pone.0253762.s006.pdf]

S1 Table: Examples for communicating uncertainty in the EMF area.

Type of uncertainty	Example
Uncertainty regarding the existence of the hazard	In 2002 the International Agency for Research on Cancer (IARC) classified static and low-frequency magnetic fields as possible causes of cancer (Group 2B) [2].<br>Source: Federal Office of Public Health FOPH (Switzerland). Fact Sheets NIR: Electric radiators (09.11.2016)<br>https://www.bag.admin.ch/bag/en/home/gesund-leben/umwelt-und-gesundheit/strahlung-radioaktivitaet-schall/elektromagnetische-felder-emf-uv-laser-licht/emf.html<br>file:///C:/Users/franc/AppData/Local/Temp/faktenblatt%20elektrischer%20radiator%20e.pdf
Uncertainty regarding the magnitude of the risk	Childhood leukaemia is a comparatively rare disease with a total annual number of new cases estimated to be 49,000 worldwide in 2000. Average magnetic field exposures above 0.3 µT in homes are rare: it is estimated that only between 1% and 4% of children live in such conditions. If the association between magnetic fields and childhood leukaemia is causal, **the number of cases worldwide that might be attributable to magnetic field exposure is estimated to range from 100 to 2400 cases per year,** based on values for the year 2000, representing 0.2 to 4.95% of the total incidence for that year. Thus, if ELF magnetic fields actually do increase the risk of the disease, when considered in a global context, the impact on public health of ELF EMF exposure would be limited.<br>Source: Maine Center for Disease Control & Prevention (Maine CDC) (November 2010), p.45<br>https://www.maine.gov/dhhs/mecdc/environmental-health/smart-meters.shtml<br>https://www.maine.gov/dhhs/mecdc/environmental-health/documents/smart-meters-review-of-government-resources-11-08-10.pdf
Uncertainty regarding risk management	**Weil aus der Forschung unterschiedlich gut abgestützte Beobachtungen vorliegen, wonach es auch noch andere als die thermischen Effekte gibt, legt die NISV zusätzlich Vorsorgewerte fest.**<br>Source: Federal Office of the Environment (FOEN): Website information from 02/23/2021: Mobile communications and 5G: dealing with adaptive antennas has been clarified (Information only available in German).<br>https://www.bafu.admin.ch/bafu/de/home/themen/elektrosmog/dossiers/bericht-arbeitsgruppe-mobilfunk-und-strahlung.html#-399594903
